# Protocol for a randomised blocked design study using telephone and text-messaging to support cardiac patients with diabetes: a cross cultural international collaborative project

**DOI:** 10.1186/1472-6963-13-402

**Published:** 2013-10-09

**Authors:** Chiung-Jung Jo Wu, Huei-Chuan Sung, Anne M Chang, John Atherton, Karam Kostner, Mary Courtney, Steven M McPhail

**Affiliations:** 1 School of Nursing, Faculty of Health, Queensland University of Technology, Victoria Park Road, Kelvin Grove QLD 4059, Australia; 2 Institute of Health and Biomedical Innovation Queensland University of Technology, Victoria Park Road, Kelvin Grove QLD 4059, Australia; 3 Department of Nursing, Tzu Chi College of Technology; Institute of Medical Sciences, Tzu Chi University, Hualien, Taiwan; 4 Taiwanese Centre for Evidence-based Health Care, Hualien, Taiwan; 5 Mater Medical Research Institute, Mater Health Services, Brisbane, Australia; 6 School of Medicine, University of Queensland, Herston QLD 4029, Australia; 7 Department of Cardiology, Royal Brisbane and Women’s Hospital, Butterfield Street, Brisbane QLD 4029, Australia; 8 Mater Health Services, Raymond Terrace, South Brisbane QLD 4101, Australia; 9 School of Nursing, Midwifery and Paramedicine, Australian Catholic University, Banyo QLD 4014, Australia; 10 Centre for Functioning and Health Research, Metro South Health, Corner of Ipswich Road and Cornwall Street, Woolloongabba QLD 4102, Australia; 11 School of Public Health and Social Work, Faculty of Health, Queensland University of Technology, Victoria Park Road, Kelvin Grove QLD 4059, Australia

**Keywords:** Cardiac, Diabetes, International collaboration, Protocol, Randomised controlled trials, Self-management, Telephone, Text-messaging

## Abstract

**Background:**

The prevalence of type 2 diabetes is rising internationally. Patients with diabetes have a higher risk of cardiovascular events accounting for substantial premature morbidity and mortality, and health care expenditure. Given healthcare workforce limitations, there is a need to improve interventions that promote positive self-management behaviours that enable patients to manage their chronic conditions effectively, across different cultural contexts. Previous studies have evaluated the feasibility of including telephone and Short Message Service (SMS) follow up in chronic disease self-management programs, but only for single diseases or in one specific population. Therefore, the aim of this study is to evaluate the feasibility and short-term efficacy of incorporating telephone and text messaging to support the care of patients with diabetes and cardiac disease, in Australia and in Taiwan.

**Methods/design:**

A randomised controlled trial design will be used to evaluate a self-management program for people with diabetes and cardiac disease that incorporates the use of simple remote-access communication technologies. A sample size of 180 participants from Australia and Taiwan will be recruited and randomised in a one-to-one ratio to receive either the intervention in addition to usual care (intervention) or usual care alone (control). The intervention will consist of in-hospital education as well as follow up utilising personal telephone calls and SMS reminders. Primary short term outcomes of interest include self-care behaviours and self-efficacy assessed at baseline and four weeks.

**Discussion:**

If the results of this investigation substantiate the feasibility and efficacy of the telephone and SMS intervention for promoting self management among patients with diabetes and cardiac disease in Australia and Taiwan, it will support the external validity of the intervention. It is anticipated that empirical data from this investigation will provide valuable information to inform future international collaborations, while providing a platform for further enhancements of the program, which has potential to benefit patients internationally.

**Trial registration:**

ACTRN 12611001196932.

## Background

Cardiac disease and type 2 diabetes are global health problems. In 2011, 17% of Australians (3.4 million) were reported to have cardiovascular disease [1] and these patients often suffer other comorbidities including type 2 diabetes [2]. The presence of both diabetes and a cardiac condition increases a patients’ likelihood of hospital readmission [1]. The prevalence of these conditions is predicted to increase, which will impact both health care expenditure and the health-related quality of life of people who develop these conditions [1,3,4].

With Westernisation, cardiac disease and diabetes have dramatically increased in Asian countries [5,6], with a similar prevalence of these diseases in Australia and in Taiwan [7,8]. The prevalence, mortality rate and healthcare cost of diabetes and cardiac disease have dramatically increased in Taiwan. In 2010, the prevalence of diabetes was 9.2% (2.11 million) in Taiwanese population [9]. Type 2 diabetes accounted for 11.5% of the total healthcare expenditure in Taiwan with most expenditure related to diabetic complications [10]. Cardiac disease was ranked as the second leading cause of death and diabetes was the fifth in 2010 [11].

With both countries experiencing the increasing economic and human consequences of these diseases in the context of finite healthcare and financial resources, innovative methods of health service delivery are required. A number of studies have demonstrated the effectiveness of self-management programs within the context of cardiac rehabilitation to assist patients to modify their lifestyle and achieve better health outcomes [12,13]. Self-management incorporating telephone and text-messages focussed on singular chronic diseases have also been associated with health benefits [4,14]. However, limited literature is available on self-management programs for patients with both cardiac disease and diabetes.

Some our previous work has investigated the use of home visits and telephone follow-up among cardiac patients with diabetes following an acute coronary care hospital admission to promote subsequent self management among this clinical population [15]. This pilot randomised trial with 20 participants demonstrated an increase in knowledge of cardiac and diabetes conditions among patients in the intervention group and supported the feasibility of this style of intervention [15]. The increasing popularity of Short-Message-Service (SMS) offers a useful addition to conventional telephone calls as a modality for communication between health professional and patients [16]. In a subsequent study, we investigated incorporating user-friendly and inexpensive SMS in addition to telephone calls to promote increased cardiac and diabetes condition knowledge among English-speaking Australians [17]. This cardiac-diabetes self-management program with telephone and SMS follow-up (T-CDSMP) increased knowledge of diabetes and cardiac conditions among patients in the intervention group [17].

Increasing condition knowledge is only one step toward changing self-management behaviours among patients with chronic conditions [18]. In addition to increasing knowledge, our T-CDSMP is intended to improve self-efficacy, self-management behaviours and subsequently health-related quality of life among patients with diabetes and cardiac conditions, as well as potentially reduce their risk of acute health events requiring hospital admission. It is envisaged that this T-CDSMP will not only be useful among English speaking patients, but also among patients from other cultural backgrounds where diabetes and cardiac conditions are similarly problematic.

### Aims

The primary aim of this investigation is to assess the short term effectiveness of the T-CDSMP on self-efficacy and self-care behaviour among patients with diabetes who also have cardiac disease in Australia and Taiwan. The secondary aim is to investigate the effect of the T-CDSMP on change in condition knowledge and health-related quality of life among these patients.

### Hypothesis

The hypothesis for the study is that the intervention group participants receiving the Cardiac-Diabetes Self-Management Program incorporating Telephone and Text-message reminders will increase their levels of self-management practice and self-efficacy more than the control group.

## Methods

### Design

**Figure 1:**
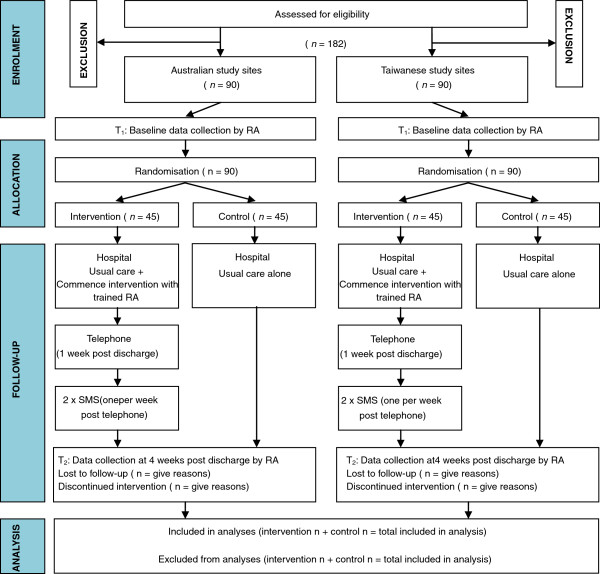
Study design: overview of data collection.

A two group, randomised block design with blinded outcome assessment will be used to address the heterogeneity of participants from differing cultural contexts [19]. Country location (Australia or Taiwan) will be the blocking variable and will ensure equal representation of patients from each country. This trial will include a follow-up of 1 month, to evaluate the short term outcomes of self-management practice and self-efficacy, as well as condition knowledge and health-related quality of life. An overview of the study design is provided in Figure 1.

### Participants

A total of 180 patients from Australia (n=90) and Taiwan (n=90) will be recruited. Patients from each country will be randomly allocated aiming for 45 patients in control and intervention groups within each location block (Figure 1). Patients will be eligible for inclusion if they have been admitted to participating coronary care units (within Australia or Taiwan) with a diagnosis of acute coronary syndrome and a diagnosis of type 2 diabetes for more than 1 year, are aged over 18 years old, have access to a telephone / device that can receive SMS, are able to read and converse in English or Chinese, and are considered medically stable by their treating clinical team. Patients will be excluded from participation in the study if they are critically ill, unconscious, or are on respiratory ventilator support. Additionally, patients who have an inpatient stay greater than 14 days will be excluded from a per-protocol analysis (as these patients will not be at home to receive the first telephone call at the scheduled time); these patients will still be included in the intention-to-treat analysis.

Eligible patients will be approached by a trained Research Assistant (RA) to obtain their individual informed consent prior to baseline data collection. Patients will be randomised to either the control or intervention groups according to the randomisation process outlined below (Figure 1). Participants in the control group will receive the usual hospital educational program comprising routine cardiac education and referral to diabetes educator if necessary. Participants in the intervention group will receive the usual hospital educational program as well as the cardiac-diabetes self-management program incorporating telephone and text-message reminders (T-CDSMP).

### Randomisation

An independent statistician, not otherwise involved in the study, will use computerised random number generation to allocate each potential participant identification number to the intervention or control group (in a one to one ratio for both locations). The allocation sequence will only be known to this person who will conceal the allocation for each participant identification number in corresponding sequentially numbered opaque envelopes. The numbered envelopes will be stored in a locked cabinet only accessible to a randomisation gatekeeper at each of the participating locations. Participants will be assigned a participant identification number in order of recruitment. After a patient has been assigned a participant identification number and completed baseline assessments, the member of the research team responsible for coordinating the interventions at the site will receive the allocation envelope from the local randomisation gatekeeper. They will then open the envelope to reveal the group allocation for the participant. Patients will not be directly told of their group allocation (in terms of intervention or control). Instead, control group participants will continue to receive their usual care, and the intervention group participants will receive the T-CDSMP in addition to their usual care.

### Intervention

Participants who are allocated to the control group will receive the usual educational program while they are in hospital. This study involves multiple sites in different countries with differing healthcare systems; therefore, the delivery of any usual care education program will be centre-oriented to represent usual care at that facility. Data pertaining to usual care in the different centres will be collected to inform conclusions that may be drawn from location specific sub-group analyses. Patients who are allocated to the intervention group will receive the usual educational programs as well as the T-CDSMP. Research staff will be trained and supervised by the research team to deliver the intervention. The T-CDSMP, developed by the research team previously [17] is based on the self-efficacy principle of Bandura’s Social Cognitive Theory [20]. The program consists of two face-to-face sessions, one telephone call (1 week post discharge) and two SMS (1 week post telephone follow-up). The program will be delivered by a research nurse (Figure 1). Strategies used will include utilising a range of information sources (mastery, vicarious experience, verbal persuasion and self-appraisal) [20] to assist patients in developing problem-solving approaches as necessary and the basic skills of monitoring their diet, physical activity, medications and blood glucose levels. Relevant case studies using case study booklets which include real stories of common scenarios from patients in Australian and Taiwanese hospitals will be discussed and patients taught other aspects of self-care such as how to recognise signs and symptoms requiring immediate medical attention.

### Data collection

There are two primary and two secondary outcomes to be measured for this study. The two primary outcomes include self-management practice and self-efficacy. The two secondary outcomes include health-related quality of life and knowledge of the conditions. Data will be collected in two episodes: at baseline (Time 1) which is after the patients’ physical condition has stabilised and they have given their consent, but before being randomised; and one month after being discharged to home (Time 2). At baseline data collection, demographic data and outcome variables will be collected. At the second data collection, primary and secondary outcomes measures will be completed.

### Measures

Questionnaires (in English and Chinese versions) will be used for collecting data at Time 1 and Time 2. Self management behaviour will assessed using the Summary of Diabetes Self-Care Activities [21,22]. Self-efficacy will be assessed using the Diabetes Management Self-Efficacy Scale (Australian-English version and Chinese version) [23,24]. Knowledge will be examined with a knowledge of condition questionnaire [25]. Health-related quality of life will be evaluated using the brief version of the World Health Organisation Quality of Life questionnaire (WHOQoL-BREF) [26,27].

### Data analysis

Prior to the commencement of data analysis, group (intervention or control) and location (Australia or Taiwan) variables will be re-coded to a pair of numbers between one and ten to which the data analyst will be blinded. In this way, the data analyst will be blinded to the group and location data code. Data analysis will be conducted with an intention-to-treat principle. Then the analysis will be repeated (for a per-protocol analysis) excluding patients with a length of stay greater than 14 days. This planned per-protocol analysis will permit an examination of the robustness of study findings to the delayed commencement of the telephone follow-up component of the intervention among the sample (due to lengths of stay longer than two weeks). While the investigators anticipate that the intention-to-treat and per-protocol analyses will return the same pattern of findings (not many participants are likely to have an acute length of stay of greater than 14-days), the per-protocol analysis will act as a sensitivity analysis with pragmatic implications for the subsequent potential translation of the intervention into health services. If the level of effectiveness of the intervention among the study sample were influenced by the exclusion or (inclusion) of patients whose follow-up telephone call was delayed due to a longer length of stay, this would have implications for potential efforts to identify patients likely to have a longer length of stay or potential future intervention delivery modifications.

Baseline demographic information of participants in each location will be outlined for the intervention and control groups using descriptive statistics. Conventional tests of hypothesis (e.g. t-tests, Mann–Whitney U, Chi-squared) will be used where indicated to examine potential differences at baseline that may require adjustments to be made when examining the influence of group (intervention or control) and location (Australia versus Taiwan) on primary and secondary outcomes. Analysis of variance / covariance will be used to examine the main effects of group (intervention or control) and location (Australia or Taiwan) as well as potential interaction between group and location for the primary and secondary outcomes. If indicated, conventional tests of hypothesis will be used to examine differences between primary and secondary outcomes across group and location. Where assumptions for parametric analyses have not been met, data will be transformed (where possible) or non-parametric alternatives will be used to make these comparisons. Data analysis will be undertaken using the Statistical Packages for the Social Sciences (SPSS) and STATA IC with a preset alpha of 0.05.

### Sample size

Sample size calculations were conducted based on conservative estimates to detect a between groups difference in primary outcomes at either location. For example, a location sample size of 90 has >80% power to detect a between groups mean difference of 11 points in the Summary of Diabetes Self-Care Activities outcome (scale range is 0 to 57) [21] and 10 points on the Diabetes Management Self-Efficacy Scale (scale range typically 62 to 200) [23], assuming standard deviations of 15.8 and 14.4 respectively, an alpha of 0.05, and exclusions due to long length of stay (or loss to follow up) of up to 28% of datasets at each site. Mean differences (and standard deviations) of this magnitude, and this potential attrition rate are more conservative than a prior intervention trial using these same outcome measures (but evaluating a clinic only based intervention) and our own pilot investigations [15,28]. Given the repeated measures nature of the data, and dual locations, this investigation will therefore have greater than 90% power to detect simple main effects of intervention (versus control) for the entire sample, and differences between location blocks (90 participants in each location).

### Ethical considerations

Ethical approvals have been sought and granted by all participating hospitals and universities in Australia and Taiwan, specifically: Royal Brisbane and Women’s Hospital Human Research Ethics Committee: Ref No: HREC/10/QRBW479, Mater Health Services Human Research Ethics Committee: Ref No:1662A, Queensland University of Technology Human Research Ethics Committee: Approval No: 1100000297, Buddhist Tzu Chi General Hospital Institutional Review Board: IRB099-112. Main ethics considerations included: 1) obtaining patients’ informed consent, highlighting that they are volunteers and could withdraw at any time; and 2) maintaining patients’ confidentiality through ensuring all information kept is non-identifiable. Data will be stored on a secure network, protected by password, and only accessed by researchers involved in this study. This investigation will be carried out in compliance with the Helsinki Declaration. This study protocol has been registered with the Australia and New Zealand Clinical Trials Registry (ACTRN12611001196932).

## Discussion

This paper has outlined a protocol for undertaking a study investigating a telephone and SMS delivered cardiac-diabetes self-management program for cardiac patients with diabetes, across two cultural contexts, measuring the outcomes of self-management behaviour, self-efficacy as well as condition knowledge and health-related quality of life. This international collaborative project aims to build on our previous T-CDSMP piloting to fulfil the needs of patients with dual cardiac and diabetes diagnoses from English and non-English speaking backgrounds. Our previous studies have provided some evidence of benefit of the T-CDSMP, particularly in the area of improving patient’s knowledge of their conditions. However the advancement of this program of research through this investigation will provide valuable empirical data to support or refute the expanded use of this program by a wider population by providing information on the impact of the program on patients from differing cultures. The potential of the program to be translated and utilised in other countries will be identified through this study, with findings providing valuable information and direction for future research to benefit patients with co-morbidities of type 2 diabetes and cardiac disease. This investigation will also form the foundation of future investigations into the medium term impacts of this intervention on health resource usage and clinical outcomes, such as hospital re-admission rates.

### Clinical implications

This study will provide clinical trial data to inform self-management programs selection for patients with cardiac disease and diabetes in different cultural contexts. It will also provide important information for nursing and medical staff seeking to incorporate remote-access communication technologies (telephone and SMS) into self-management programs for patients with dual conditions of cardiac disease and diabetes. These patients with dual diagnoses have poorer health outcomes and complex management requirements. Current models of health care tend to compartmentalise management with increasingly specialised care for different diseases. However, this is in direct contrast to the reverse trend of chronic disease with increasing numbers of aging patients with multiple comorbidities. Our program seeks to allow the patient to take a more active role in the process of harmonising care across different specialties. Furthermore, whilst our program is focussed on diabetes and cardiac disease, it may nonetheless provide a model of care which may be transferrable to other chronic diseases.

### Limitations

There were challenges experienced when planning this study protocol, including understanding the variations in research processes, healthcare personnel knowledge and skill across two countries. Strategies for ensuring data collection procedures and intervention delivery will be consistent and rigorous, required substantial time, significant planning and clear communication strategies amongst all research team members. A limitation of this investigation is that it does not have scope to assess medium term impacts on health care resource usage and clinical outcomes, such as hospital readmission rates.

## Conclusion

This proposed international, collaborative, intervention study across two differing cultures using a randomised controlled trial design (with country as a blocking variable), will provide valuable information on cultural variations that may need to be addressed to improve delivery of self-management programs that incorporate telephone and text messaging follow-up. Investigating whether this program incorporating inexpensive, user friendly remote-access communication technologies to meet the needs of diabetes patients with acute coronary syndrome across two diverse countries will also provide insights into the potential benefit of expanding these techniques for use in other similar programs globally.

## Competing interests

The authors declare that they have no competing interests.

## Authors’ contributions

C-JW contributed to idea conception, study design, coordination of study preparation (Australia), data analysis plan, principal drafting of this protocol, as well as appraisal, editing and drafting of protocol revisions. H-CS contributed to study design, study preparation (Taiwan) and protocol appraisal. AC, JA, KK, and MC contributed to study design and appraisal of the protocol. SMM contributed to study design, appraisal of the protocol, drafting of revisions and data analysis plan. All authors read and approved the final manuscript.

## Pre-publication history

The pre-publication history for this paper can be accessed here:

http://www.biomedcentral.com/1472-6963/13/402/prepub
